# Amyloid deposition and ApoE4 carriers in idiopathic normal pressure hydrocephalus

**DOI:** 10.1186/2045-8118-12-S1-P5

**Published:** 2015-09-18

**Authors:** Masahiko Bundo, Akinori Nakamura, Takashi Kato, Shunpei Niida, Kaori Iwata, Chihomi Sawado, Kengo Ito

**Affiliations:** 1 National center for geriatrics and gerontology, Japan

## Introduction

It is known that idiopathic normal pressure hydrocephalus (iNPH) can co-morbid with Alzheimer disease (AD). Previous studies, probing amyloid (Aβ) deposition by cortical biopsy during a ventriculo-peritoneal shunt, have shown that Aβ deposition was found in 40~45 % of iNPH patients. However, the reason for this high prevalence is not well understood. Therefore, the objective of this study was to investigate whether the prevalence of the Aβ deposition in iNPH is explainable by a simple overlap of the AD pathology in general population, or is modified by some specific effects of iNPH.

## Methods

In this study, 11C-Pittsburgh compound B (11C-PiB) PET and Apolipoprotein E genotype were examined in age- matched 70 Cognitively Normal Elderly (CNE), 19 Alzheimer disease (AD) and 31 probable iNPH patients, to investigate the risk of co-morbidity of AD in iNPH patients. The chi-square analysis was used for the statistical analysis. The Bonferroni correction was used for the multiple comparisons.

## Results

Amyloid deposition was shown in 19.0% of CNE, 81.3% of AD, and 52.0% of iNPH, while ApoE4 carriers were found in 19.0% of CNE, 73.7% of AD, and 29.0 of iNPH. The rate of amyloid deposition of iNPH was significantly higher than CNE (p < 0.005) and lower than AD (p < 0.005). On the other hand, the probability of the apoE4 carriers in iNPH was significantly lower than AD (p < 0.005), but did not show any significant difference from CNE (p = 0.56).

## Conclusions

In iNPH, the rate of amyloid deposition is higher than CNE, while there were no differences in the probability of the ApoE4 carriers. These results suggest that iNPH patients may have some mechanisms to facilitate the amyloid deposition.

